# FOLFOXIRI/Bevacizumab Plus Nivolumab as First-Line Treatment in Metastatic Colorectal Cancer RAS/BRAF Mutated: Safety Run-In of Phase II NIVACOR Trial

**DOI:** 10.3389/fonc.2021.766500

**Published:** 2021-12-14

**Authors:** Angela Damato, Francesca Bergamo, Lorenzo Antonuzzo, Guglielmo Nasti, Francesco Iachetta, Alessandra Romagnani, Erika Gervasi, Mario Larocca, Carmine Pinto

**Affiliations:** ^1^ Medical Oncology Unit, Azienda USL (Unità Sanitaria Locale)- IRCCS (Istituto di Ricovero e Cura a Carattere Scientifico) di Reggio Emilia, Reggio Emilia, Italy; ^2^ Department of Medical Biotechnologies, University of Siena, Siena, Italy; ^3^ Medical Oncology Unit 1, Clinical and Experimental Oncology Department, Veneto Institute of Oncology Istituto Veneto Oncologico (IOV) - Istituto di Ricovero e Cura a Carattere Scientifico (IRCCS), Padua, Italy; ^4^ Clinical Oncology Unit, Careggi University Hospital, Department of Experimental and Clinical Medicine, University of Florence, Florence, Italy; ^5^ Medical Oncology, Abdominal Department, National Cancer Institute G. Pascale Foundation, Naples, Italy

**Keywords:** safety run-in, nivolumab, FOLFOXIRI, bevacizumab, colorectal cancer, RAS mutation, BRAF mutation

## Abstract

The NIVACOR trial is a phase II study assessing the efficacy and safety of nivolumab in combination with FOLFOXIRI/bevacizumab in first-line setting in patients affected by metastatic colorectal cancer (mCRC) RAS/BRAF mutated. We report safety run-in results in the first 10 patients enrolled. Patients received triplet chemotherapy with FOLFOXIRI scheme plus bevacizumab, in association with nivolumab every 2 weeks for 8 cycles (induction phase) followed by bevacizumab plus nivolumab every 2 weeks (maintenance phase), until progression of disease or unacceptable toxicities. The first ten patients were evaluated: 7 experienced at least one adverse event (AE) related to FOLFOXIRI/bevacizumab and 2 related to nivolumab. The most frequent grade 1–2 AEs related to FOLFOXIRI/bevacizumab were diarrhea and fatigue (71%), nausea and vomiting (57%); 3 (43%) had grade 3–4 neutropenia, and 2 (20%) patients developed grade 1–2 AEs nivolumab related: skin rash and salivary gland infection. Two patients delayed the dose because of serious AEs, proteinuria and salivary gland infection; one patient discontinued experimental treatment due to the ileo-urethral fistula and concurrent *Clostridium* infection diarrhea. No treatment- related death occurred. The safety run-in analysis of NIVACOR trial reassured using co-administration of FOLFOXIRI/bevacizumab and nivolumab was well tolerated with an acceptable toxicity profile.

**Clinical Trial Registration:**

https://clinicaltrials.gov/, (NCT04072198).

## Introduction

Colorectal cancer is the fourth commonly diagnosed cancers and among the leading cause of cancer-related deaths globally (1). Around 40–50% of cases are diagnosed in advanced stages with a median overall survival (OS) increased over the 30 months and a 5-year survival rate of approximately 12% (1, 2). The backbone of treatment in first-line in mCRC comprises chemotherapy scheme combination (5-fluorouracil, oxaliplatin, irinotecan) combined with specific monoclonal antibody anti-epidermal growth factor receptor (EGFR), cetuximab or panitumumab, or anti-vascular endothelial growth factor (VEGF), such as bevacizumab (3, 4).

The antibodies’ choice depends on RAS mutation occurrence, which represent 50-55% of cases in mCRC. Therefore, BRAF V600E mutation represented the additional prognostic negative factor in mCRC occurring in 5–21% of cases (5–10), although recently there has been published a randomized, phase III trial of combination with an anti-BRAF inhibitor (encorafenib), cetuximab, and an anti-MEK inhibitor (binimetinib), in patients with mCRC BRAF V600E mutated, after one or two previous regimens, resulting in significantly longer OS and a higher response rate than standard therapy (irinotecan plus cetuximab) (11).

The TRIBE phase III trial demonstrated a superiority of FOLFOXIRI plus bevacizumab compared to FOLFIRI plus bevacizumab in the first-line setting in mCRC in terms of progression-free survival (PFS) (12.3 *versus* 9.7 months, hazard ratio 0.77, 95% CI 0.65–0.93; p = 0.006), also in BRAF mutated subgroup (12, 13). Selecting the best upfront therapy is crucial in the mCRC to warrant a better survival and response to treatment. For these reasons, the intensification of the chemotherapy backbone in previously untreated mCRC patients is recommended.

Immune checkpoint inhibitors (ICIs) have announced new opportunities in cancer therapy (14–17). Programmed death 1 (PD-1) blockade has clinical benefit in MSI-H or dMMR mCRC after previous therapies. Indeed, the FDA approved nivolumab and pembrolizumab for patients with MSI-H/dMMR mCRC evolved after treatments with fluoropyrimidine, oxaliplatin, and irinotecan (18–20). Recently, the KEYNOTE-177 phase III trial revealed that front-line pembrolizumab was superior to chemotherapy in terms of PFS (16.5 *versus* 8.2 months, hazard ratio, 0.60; 95% CI, 0.45 to 0.80; P=0.0002). The overall response (complete or partial response) was observed in 43.8% of patients (95% CI, 35.8 to 52.0) in the pembrolizumab arm compared to 33.1% (95% CI, 25.8 to 41.1) in the chemotherapy arm; the complete response of disease was observed in 11 and 4% of patients, respectively. Among these, 83% of patients had ongoing responses in the pembrolizumab arm (21).

The sensitivity to immune checkpoint inhibitors in MSI-H/dMMR mCRC is reasonably due to a more efficient immune background linked to a high burden of neoantigens arising from the hypermutated tumor cells, able to prompt a compelling immune response. Conversely, MSS/pMMR tumors are mainly immune deprived or immune desert, because of a low or deficient T-cell infiltration and reduced expression of checkpoint proteins (22). To recruit stimulated immune cells in the tumor microenvironment, combination strategies are investigated including immunotherapy and other drugs with immunomodulatory features, such as dual immune checkpoint blockade, radiotherapy, chemotherapy, or other targeted therapies. Furthermore, there is a rising effort to detect different biomarkers to select patients most likely responsive to immunotherapy and to recognize possible resistance mechanisms.

The NIVACOR study was designed to assess the efficacy and safety of nivolumab in combination with chemotherapy triplet scheme (FOLFOXIRI) plus bevacizumab in first-line treatment in patients affected by mCRC RAS/BRAF mutated, regardless of the microsatellite status. A preliminary safety analysis was planned after the 10th patient enrolled to detect early and acute toxicity. We present the safety run-in results of phase II NIVACOR trial.

## Materials and Methods

### Study Design and Population

**Figure 1:**
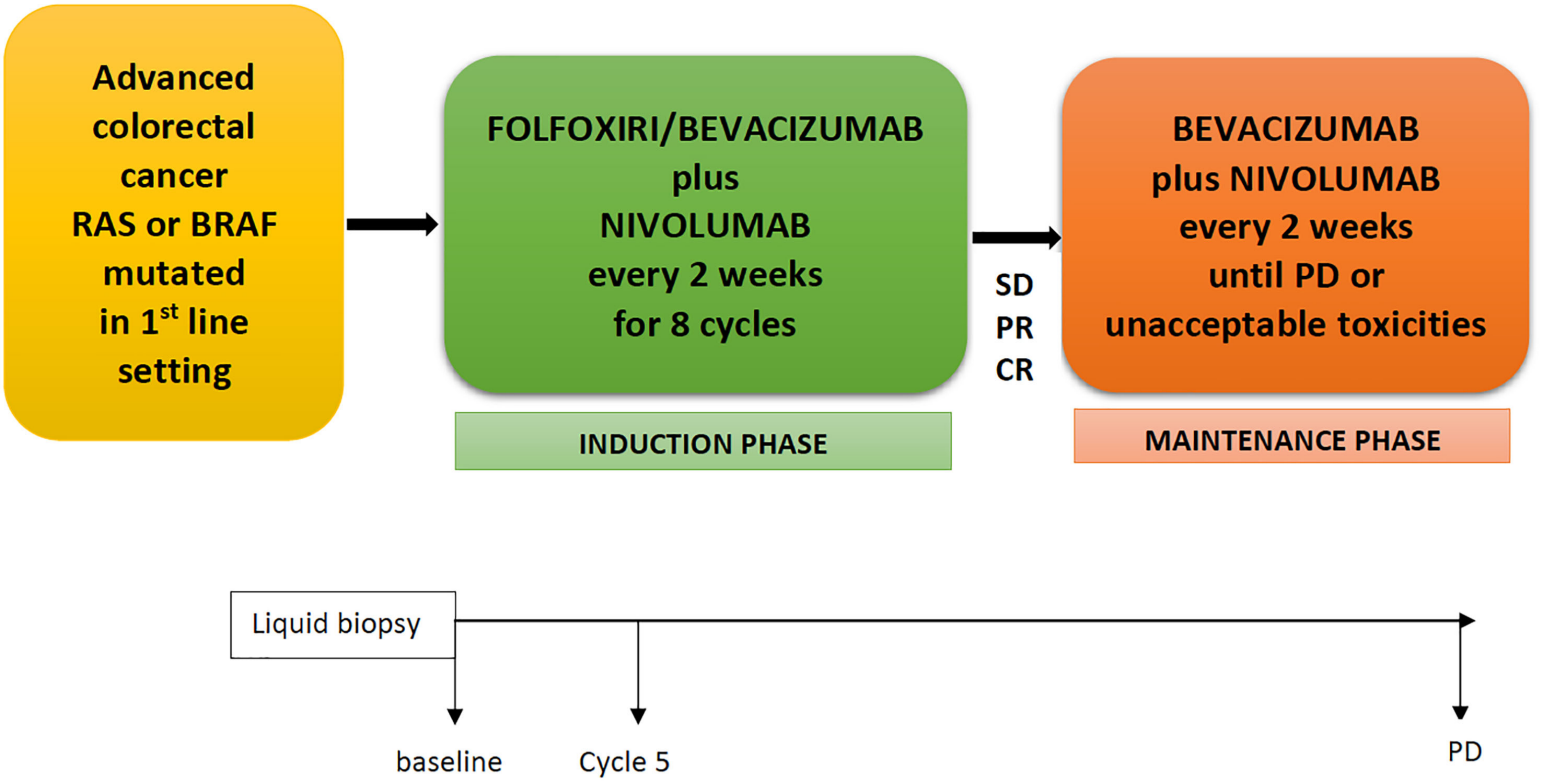
NIVACOR Study design. FOLFOXIRI, 5-fluorouracil, irinotecan, oxaliplatin; SD, stable disease; PR, partial response; CR, complete response; PD, progression disease.

The NIVACOR (NCT04072198) is a single arm, open-label, multicenter, phase II study with a safety run-in phase. Patients with mCRC RAS/BRAF mutated in the first-line setting received nivolumab in combination with a triplet of chemotherapy, FOLFOXIRI, plus bevacizumab for eight cycles (induction phase) and subsequently, in patients with partial response or control of disease, nivolumab and bevacizumab until progression of disease or unacceptable toxicities (maintenance phase) (**Figure 1**). Collateral studies are also in process; liquid biopsy will be performed at baseline, before cycle 5, and at disease progression.

Patients eligible for the enrolment require a histological diagnosis of adenocarcinoma, mutation of RAS or BRAF confirmed, advanced and unresectable disease untreated with previous chemotherapy for metastatic disease. All inclusion and exclusion criteria can be available in the study protocol (**Table S1**).

### Study Objectives and Endpoints

The trial was designed to assess the efficacy of nivolumab in addition to FOLFOXIRI plus bevacizumab in terms of response rate. The primary endpoint was the objective response rate (ORR) evaluated by the investigator assessment according to RECIST 1.1 criteria. Secondary objectives were safety profile according to Common Terminology Criteria for Adverse Events (CTCAE) v.4.03, overall survival (OS), time to progression (TTP), duration of response (DoR), and quality of life evaluated with the EORTC QLQ-C30.

### Treatment Scheme and Modalities

All eligible patients receive 8 cycles of nivolumab in combination with FOLFOXIRI/bevacizumab every 2 weeks (induction phase), followed by nivolumab plus bevacizumab every 2 weeks in patients that achieved partial response, complete response, or stable disease. The treatment will be continued until unacceptable toxicity, disease progression, or patient/physician’s decision. All drugs are administered intravenously. Bevacizumab at a dose of 5 mg/kg every 2 weeks; nivolumab at flat dose of 240 mg every 2 weeks. FOLFOXIRI scheme will be administered as irinotecan infusion at 165 mg/m2 for 60 min (min), followed by oxaliplatin infusion at 85 mg/m2 simultaneously with leucovorin 200 mg/m2 for 120 min, and lastly continuous infusion for 48 h of 5-fluorouracil at dose of 3,200 mg/m2.

### Treatment Evaluation

Treatment visits were performed on day 1 (+3 days) of each cycle with vital signs, a physical examination, hematology and biochemical laboratory analysis, AEs description, and concomitant treatments report. Treatment efficacy was evaluated by the investigator according to Response Evaluation Criteria in Solid Tumours (RECIST) 1.1 criteria every 8 weeks during investigational treatment, and subsequently every 3 months for 3 years.

### Safety Run-In Analysis

Considering the safety profile of nivolumab in combination with FOLFOXIRI/bevacizumab has not been evaluated in mCRC patients so far, a safety run-in phase was achieved. This analysis was arranged to evaluate the feasibility of the investigational treatment by noticing the first ten patients enrolled. In order to be able to describe the safety profile, an Independent Monitoring Committee (IDMC) reviewed data 28 days after the 10th patient included. At least one cycle of experimental treatment had been administered. Grading of adverse events (AEs) was collected according to the Common Terminology Criteria for Adverse Events (CTCAE) version 4.03. The IDMC provided an endorsement as to whether the study may resume, whether amendments to the protocol should be implemented, or whether the study should be closed. If the experimental treatment study was judged feasible and no major safety concerns arose, the enrollment would be resumed and involved further participating sites.

### Statistical Analysis for Primary Endpoint

The sample size was estimated using the A’Hern (23) variation of the original Fleming (24) one-stage design. The study needs 64 patients to decide whether P < 0,66 or > 0,80. An overall 70 patients should be enrolled supposing 10% of patients’ discontinuation due to unacceptable toxicity or non-compliance to the treatment. Our hypothesis is that nivolumab in addition to FOLFOXIRI/bevacizumab increases the overall response rate (ORR) from 66 to 80%, the latter considered sufficiently valuable to pursue this experimental combination in a phase III trial.

## Results

### Patient Characteristics

Between October 2019 and June 2020, a safety run-in phase was conducted at the study Coordinating Center. Ten patients were enrolled and received experimental study treatment. According to the study protocol, IDMC reviewed the safety data when 10 patients had received at least 1 cycle of the study treatment. The median age of patients was 58 (range 32–66), and 6 patients (60%) were male. The median cycles of treatment were 5.5 (range 1–9). Nine (90%) patients had Eastern Cooperative Oncology Group (ECOG) performance status equal to 0. All of patients (100%) were KRAS G12D mutated and BRAF wild type; 2 (2%) patients had MSI-H/dMMR. Patient characteristics and baseline demographics are summarized in **Table 1**.

**Table 1:** Patient’s characteristics.

Variables	All patients (n = 10)
**Clinical characteristics**	
Age (years)	58 (32–66)
Gender (n, %)	
Male	6 (60)
Female	4 (40)
Performance status (n, %)	
0	9 (90)
1	1 (10)
**Tumor characteristics**	
Primary tumor site (n, %)	7 (70)
Left	0 (0)
Transverse	3 (30)
Right	
Site of metastases (n, %)	
Liver	4 (40)
Lung	2 (20)
Peritoneum	4 (40)
Lymph nodes	2 (20)
Others	2 (20)
**Molecular alterations (n, %)**	
RAS mutation	10 (100)
KRAS G12D	10 (100)
NRAS	0 (0)
BRAF mutation	0 (0)
MSS	8 (80)
MSI-H	2 (20)

MSS, microsatellite stable; MSI-H, microsatellite instability high.

### Adverse Events in Overall Population

Adverse events (AEs) are summarized in **Table 2**.

**Table 2:** Adverse events in whole population (n = 10).

Adverse Events	FOLFOXIRI/BEV	FOLFOXIRI/BEV	Nivolumab	Nivolumab	SAE
	Grade 1/2	Grade 3/4	Grade 1/2	Grade 3/4	
	n (%)	n (%)	n (%)	n (%)	n (%)
Patients exhibit at least one toxicity	7 (100)	7 (100)	2 (100)	0 (0)	4 (100)
**Gastrointestinal toxicities**					
**Nausea**	4 (57)	1 (14)	0 (0)	0 (0)	
**Vomiting**	4 (57)	0 (0)	0 (0)	0 (0)	
**Diarrhea**	5 (71)	0 (0)	0 (0)	0 (0)	1 (25)
**Fatigue**	5 (71)	1 (14)	0 (0)	0 (0)	
**Others**	5 (71)	0 (0)			
**Hematological toxicities**					
**Neutropenia**	1 (14)	3 (43)	0 (0)	0 (0)	
**Febrile neutropenia**	0 (0)	1 (14)	0 (0)	0 (0)	
**Anemia**	2 (29)	0 (0)	0 (0)	0 (0)	
**Infusion-related toxicity**	1 (14)	0 (0)	0 (0)	0 (0)	
**Paresthesia**	1 (14)	0 (0)	0 (0)	0 (0)	
**Skin rash**	2 (29)	1 (14)	1 (50)	0 (0)	
**Fatigue**	5 (71)	1 (14)	0 (0)	0 (0)	
**Salivary gland infection**	0 (0)	0 (0)	1 (50)	0 (0)	1 (25)
**Conjunctivitis**	1 (14)	0 (0)	0 (0)	0 (0)	
**Ileo-urethral fistula**	0 (0)	0 (0)	0 (0)	0 (0)	1 (25)
**Proteinuria**	1 (14)	0 (0)	0 (0)	0 (0)	1 (25)

BEV, bevacizumab; SAE, serious adverse event.

Among all (N=10, 100%), 70% (N=7) of patients experienced at least one AE related to FOLFOXIRI plus bevacizumab, and 20% (N=2) developed immune-related AEs (irAEs) to nivolumab. The most frequent grade 1–2 AEs related to FOLFOXIRI plus bevacizumab were diarrhea (71%, N=5), fatigue (71%, N=5), nausea (57%, N=4), and vomiting (57%, N=4). Instead, grade 1–2 skin rash (50%, N=1) and salivary gland inflammation (50%, N=1) occurred as irAEs. One patient (14%) developed recurrent infusion-related toxicity to oxaliplatin during the 1^st^ and 2^nd^ cycle of treatment, leading to permanent drug discontinuation. Concerning grade 3–4 AEs, 43% (N=3) of patients developed neutropenia, and N=1 (14%) patient had febrile neutropenia associated to FOLFOXIRI plus bevacizumab. Additionally, 1 (14%) patient developed a G3/4 skin rash, which was attributed to toxicity of chemotherapy and possibly to oxaliplatin but did not appear during intravenous infusion but approximately 6 h after administration of the 2^nd^ and 3^rd^ cycle of treatment. No one patient exhibited grade 3–4 irAEs. Regarding the serious AEs (SAEs), 4 of them were reported: 1 (25%) patient revealed proteinuria associated to bevacizumab, resulting in the treatment delay; 1 (25%) patient developed salivary gland infection possibly related to nivolumab, treated with non-steroidal anti-inflammatory drug (ketoprofen) and pain relievers; and 1 patient had ileo-urethral fistula (25%) and concurrent diarrhea secondary to *Clostridium difficile* infection (25%) after the first cycle of FOLFOXIRI/bevacizumab plus nivolumab, leading to colostomy packaging and permanent treatment discontinuation. Despite this, the IDMC judged the study treatment combination tolerable and feasible and worthy of further investigation. The enrollment resumed and currently is concluded at 73 patients enrolled in 11 Italian participating sites.

### Outcome of Patients Discontinued

Overall, 1 patient discontinued treatment for SAEs. After a median follow-up of 17 months, 4 out of 10 patients still remained in the experimental treatment.

## Discussion

In the NIVACOR study, the most common toxicities reported for the experimental combination were those anticipated with FOLFOXIRI plus bevacizumab alone, as reported in the TRIBE study (12, 13).

Several clinical trials exploiting combination of chemotherapy with immune-checkpoint inhibitors have been published. Among these, we discuss studies using chemotherapy regimen with fluorouracil, oxaliplatin, and irinotecan (**Table 3**).

**Table 3:** Most common treatment-related adverse events with immunotherapy and chemotherapy combinations in Phase II and III trials.

	Phase	Primary tumor site	Study population (N=)	Therapeutic regimens	AEs grade 1–2 (%)	AEs grade 3–4 (%)
**Khemka V et al.** (25)	Phase I/II	Colon	8	Nivolumab Capecitabine Irinotecan	Fatigue^*^	–
					Nausea^*^	
					Diarrhea^*^	
**Rogers JE et al.** (26)	Retrospective	Gastroesophageal	15	Nivolumab	Fatigue^*^	Febrile neutropenia^*^
				Capecitabine or Fluorouracil	Hypothyroidism^*^	Liver function enzyme alteration^*^
				Irinotecan		
**Moheler M et al.** (27)	Phase III	Gastric	468	Nivolumab	Nausea, diarrhea, and peripheral neuropathy (≥25%)	Cerebrovascular accident, febrile neutropenia, gastrointestinal inflammation, and pneumonia (<1%)
		Gastroesophageal junction		FOLFOX^a^ or CAPOX^b^		
		Esophageal				
**Boku N et al.** (28)	Phase II	Gastric	39	Nivolumab +/−	–	Neutropenia (14.3 *vs* 16.7%)
		Gastroesophageal junction		S-1/oxaliplatin or Nivolumab +/−		Peripheral neuropathy, anemia, and nausea (11.1% for each)
				Capecitabine/		
				Oxaliplatin		
**Kawazoe A et al.** (29)	Phase IIb	Gastric	54	Pembrolizumab	–	Platelet count decreased (14.8%)
		Gastroesophageal junction		S-1		Neutrophil count decreased (13.0%)
				Oxaliplatin		Colitis and adrenal insufficiency (5.6%)
**Bang YJ et al.** (30)	Phase II	Gastric	25	Pembrolizumab Cisplatin	–	Neutropenia (48%)
		Gastroesophageal junction	(Cohort 2)	5-fluorouracil		Stomatitis (20%)
						Anemia, platelet count decreased, fatigue, and maculopapular rash (8%)
**Antoniotti C et al.** (31)	Phase II	Colon	6	Atezolizumab	–	Neutropenia, diarrhea, and hypertension (16.7%)
			(Arm B)	FOLFOXIRI^c^		
				Bevacizumab		

*Percentages not specified for this event; AEs, adverse events; a, fluorouracil, folinic acid, oxaliplatin; b, capecitabine, oxaliplatin; c, fluorouracil, folinic acid, irinotecan, oxaliplatin.

Khemka et al. conducted a phase Ib/II study on colorectal cancer and pancreatic adenocarcinoma patients to evaluate safety and clinical activity of nivolumab plus an oral fluoropyrimidine, capecitabine, and irinotecan (CAPIRI scheme) (25). Patients were treated with nivolumab 3 mg/kg on day 1 and day 15 every 28 days cycle along with CAPIRI until disease progression or toxicity. The most common grade 1–2 AEs were fatigue, nausea, and diarrhea in 50% of patients; no infusion-related reactions were observed. There were no dose-limiting toxicities or SAEs reported. The authors suggested the following dosing scheme for the phase 2 section of the trial: nivolumab 3 mg/kg days 1 and 15, irinotecan 175 mg/m2 day 1 and 15, capecitabine 1,000 mg twice daily 5 days on and 2 days off every week.

In a retrospective analysis, 15 patients affected by advanced gastroesophageal cancers treated with nivolumab 240 mg flat dose, irinotecan 120 mg/m2, and 5-FU 2,000 mg/m2 over 46 h (or capecitabine 1,250 mg/m2/day for 7 days on/7 days off) given every 2 weeks. One patient had febrile neutropenia, and 1 patient grade 3 liver function enzyme alteration. Most patients had grade ≤2 AEs, especially fatigue (26).

The CheckMate 649, the largest phase III randomized trial, displayed the first results comparing two arms of treatment, nivolumab plus chemotherapy (FOLFOX or XELOX) *versus* chemotherapy alone, in patients affected by advanced gastric/gastroesophageal junction/esophageal cancer. The most common any grade TRAEs (≥25%) across both arms were nausea, diarrhea, and peripheral neuropathy. Regarding TRAEs with potential immunologic etiology, grade 3–4 TRAE events occurred in ≤5% of patients, and there were no grade 5 events. The incidence of TRAEs in patients whose tumors expressed PD-L1 CPS ≥ 5 was consistent with all treated patients across both arms (27).

The safety of nivolumab added to S-1 plus oxaliplatin (SOX) or capecitabine plus oxaliplatin (CapeOX) as first-line therapy for advanced or recurrent HER2-negative gastric or esophageal-gastric cancer was evaluated in the part 1 of the ATTRACTION-4 study. Patients were randomized to receive nivolumab flat dose of 360 mg every 3 weeks, S-1 40 mg/m2 orally twice daily for 14 days and 7 days off (or capecitabine 1,000 mg/m2 orally twice daily for 14 days and 7 days off) plus oxaliplatin 130 mg/m2 on day 1 every 3 weeks, until disease progression or unacceptable toxicity. Among 40 patients enrolled, 38 included the safety and efficacy analysis. Most of grade 3–4 AEs were neutropenia (14.3%) in the nivolumab plus SOX group, while in CapeOX group described neutropenia (16.7%), followed by anemia, nausea, and neurological toxicities in 11.1% of cases, respectively. No treatment-related death occurred. Discontinuations due to treatment-related adverse events (TRAEs) were reported in five patients (28).

The safety of pembrolizumab combined to S-1 and oxaliplatin (SOX) as the first-line therapy in Japanese patients affected by advanced gastric or gastroesophageal junction cancer was evaluated in a phase IIb KEYNOTE-659 study. Grade >3 TRAEs were reported in 57.4% of patients. The most common grade >3 TRAEs were platelet and neutrophil counts decreased (14.8 and 13.0%), colitis and adrenal insufficiency in 5.6% of cases. No treatment-related deaths occurred (29).

Furthermore, in the KEYNOTE-059 trial, 25 patients with advanced gastric or gastroesophageal junction cancer received pembrolizumab in combination with cisplatin and continuous infusion of 5-fluorouracil. Grade 3 TRAEs occurred in 15 patients (60%), and 4 (16%) experienced grade 4 neutropenia. Pembrolizumab-related AEs were observed in 12 (48.0%) patients; grade 3 stomatitis and maculopapular rash occurred in 2 (8%) patients and 1 (4%) patient, respectively (30).

Recently, the ongoing phase II AtezoTribe study was published. Patients with mCRC, regardless of microsatellite status, were randomized 1:2 in first-line therapy to receive FOLFOXIRI plus bevacizumab (arm A) or FOLFOXIRI plus bevacizumab and atezolizumab (arm B) up to 8 cycles, followed by maintenance with 5-FU/leucovorin plus bevacizumab with or without atezolizumab until disease progression or unacceptable toxicity. A safety run-in phase was conducted in the first 8 patients enrolled (2 in arm A and 6 in arm B). No grade 4 or SAEs were described. Grade 3 AEs that emerged were neutropenia for 1 patient in arm A and 1 patient in arm B; diarrhea and hypertension were experienced in 1 patient in arm A and 1 patient in arm B. This study was judged safe, and the enrollment has been resumed and currently completed (31).

In line with the literature data, the main grade 1–2 AEs related to chemotherapy and antiangiogenetic drug affect mainly the gastrointestinal and hematological toxicity, such as diarrhea and fatigue (71%), nausea and vomiting (57%), followed by grade 3–4 neutropenia (43%) and febrile neutropenia (14%). Grade 1–2 immune-related AEs were reported in two patients as described above (skin rash and salivary gland infection). Two SAEs have been reported but both related to FOLFOXIRI and bevacizumab. Overall, these toxicities appear to be manageable.

## Conclusions

The safety run-in results of the NIVACOR trial do not raise concerns regarding the co-administration of chemotherapy (FOLFOXIRI), an anti-VEGF antibody (bevacizumab), and an anti-PD-L1 antibody (nivolumab). Although the analysis concerns only the first ten patients enrolled, the study resumed and the enrolment on March 1, 2021, was concluded. Data on the primary and secondary endpoints will be presented in the final analysis, including comprehensive safety analysis across overall population.

These preliminary results support the clinicians’ decision to continue the phase II NIVACOR trial to evaluate the efficacy of the association between immune checkpoint agent with a triplet chemotherapy and anti-VEGF inhibitor in mCRC RAS/BRAF mutated, regardless of microsatellite status.

## Data Availability Statement

The datasets presented in this study can be found in online repositories. The names of the repository/repositories and accession number(s) can be found in the article/**Supplementary Material**.

## Ethics Statement

The studies involving human participants were reviewed and approved by Agenzia Italiana del Farmaco—AIFA) on February 8, 2019 (Protocol version 2.0, January 14th 2019), and an Independent Ethics Committee (Area Vasta Emilia Nord). The patients/participants provided their written informed consent to participate in this study.

## Author Contributions

Conceptualization: AD, CP, and FI. Methodology: AD and CP. Formal analysis: AD and CP. Investigation: AD, CP, FI, FB, LA, GN, and ML. Data curation: AD, CP, AR, and EG. Writing—original draft preparation: AD. Writing—review and editing: AD, CP, FI, LA, and AR. Visualization: AD, CP, FI, FB, LA, GN, ML, AR, and EG. Supervision: AD and CP. Project administration: AD. All authors contributed to the article and approved the submitted version.

## Funding

This research received an unrestricted grant by Bristol Meyer Squibb Italy.

## Conflict of Interest

CP reports outside the submitted work personal fees for advisory role, speaker engagements, and travel and accommodation expenses from Amgen, Astellas, Astra-Zeneca, Bayer, Bristol Meyer Squibb, Clovis Oncology, Ipsen, Janssen, Incyte, Merck-Serono, Merck Sharp and Dohme, Novartis, Roche, and Sanofi.

The remaining authors declare that the research was conducted in the absence of any commercial or financial relationships that could be construed as a potential conflict of interest. The funders had no role in the design of the study; in the collection, analyses, or interpretation of data; in the writing of the manuscript, or in the decision to publish the results.

## Publisher’s Note

All claims expressed in this article are solely those of the authors and do not necessarily represent those of their affiliated organizations, or those of the publisher, the editors and the reviewers. Any product that may be evaluated in this article, or claim that may be made by its manufacturer, is not guaranteed or endorsed by the publisher.
