# Supplementary material for: FOLFOXIRI/Bevacizumab Plus Nivolumab as First-Line Treatment in Metastatic Colorectal Cancer RAS/BRAF Mutated: Safety Run-In of Phase II NIVACOR Trial
Source: Front Oncol. 2021 Dec 14;11:766500. doi: 10.3389/fonc.2021.766500 (PMC8712943; doi:10.3389/fonc.2021.766500)
Supplement: Supplementary file 1 [file Table_1.doc]

**Table S1.** Principal inclusion and exclusion criteria of NIVACOR trial

| **Inclusion criteria** | **Exclusion criteria** |
| --- | --- |
| 1) Written informed consent.  2) Male or female of 18-75 years of age on day of signing informed consent.  3) Histologically confirmed diagnosis of colorectal cancer RAS/BRAF mutated.  4) Initially unresectable metastatic colorectal cancer not previously treated with chemotherapy for metastatic disease.  5) Patients suitable for first line chemotherapy.  6) Life expectancy > 3 months.  7) At least one site of measurable disease per RECIST criteria.  8) Performance status of 0-1 on the ECOG Performance Scale.  9) Adequate organ function, defined as  a) Neutrophils >1.5 x 10^9^/L,  b) Platelets >100 x 109/L,  c) Hemoglobin >9 g/dl  d) Total bilirubin < 1.5 time the upper-normal limits (UNL) of the normal values and ASAT (SGOT) and/or ALAT (SGPT) <2.5 x UNL (or <5 x UNL in case of liver metastases)  e) Alkaline phosphatase <2.5 x UNL (or <5 x UNL in case of liver metastases)  f) Creatinine clearance >50 mL/min or serum creatinine < 1.5 x UNL  g) Urine dipstick of proteinuria <2+. Patients discovered to have 2+ proteinuria on dipstick urinalysis at baseline, should undergo a 24-hour urine collection and must demonstrate <1 g of protein/24 hr.  10) Availability at baseline of a representative formalin-fixed, paraffin-embedded (FFPE) diagnostic tumor specimen, as primary and/or metastatic tumor tissue block or as fifteen 5-micron unstained slides are allowed (the neoplastic cell content of each tumor sample will be assessed and in those cases with neoplastic cells <50% a macro-dissection of the specimen will be performed, if possible).  11) If DPD status is known it must be wild type. No restrictions are applied if DPD status in unknown.  12) Women of childbearing potential must have a negative blood pregnancy test within 24 hours prior to the start of study drug. For this trial, women of childbearing potential are defined as all women after puberty, unless they are postmenopausal for at least 12 months, are surgically sterile, or are sexually inactive.  13) Subjects and their partners must be willing to avoid pregnancy during the trial and until 5 months for WOCBP (Women of Childbearing Potential) and 7 months for male subjects with female partners of WOCBP after the last trial treatment. Male subjects with female partners of childbearing potential and female subjects of childbearing potential must, therefore, be willing to use adequate contraception as approved by the investigator (barriers contraceptive measure or oral contraception). | 1) Prior chemotherapy, excluded patient treated in neo/adjuvant setting at least 12 months before diagnosis of metastatic disease.  2) Radiotherapy to any site within 4 weeks before the study.  3) Serious, non-healing wound, ulcer, or bone fracture.  4) Evidence of bleeding diathesis or coagulopathy.  5) Uncontrolled hypertension and prior history of hypertensive crisis or hypertensive encephalopathy.  6) Systemic corticosteroids within 2 weeks of the first dose of nivolumab.  7) Diagnosis of immunodeficiency or is receiving systemic steroid therapy or any other form of immunosuppressive therapy within 14 days prior to the first dose of trial treatment.  8) Additional malignancy in the last 5 years. Exceptions include basal cell carcinoma of the skin, squamous cell carcinoma of the skin, or in situ cervical cancer that has undergone potentially curative therapy.  9) Active and untreated brain (CNS) metastases and/or carcinomatous meningitis. Subjects with previously treated brain metastases may participate provided they are not using steroids for at least 7 days prior to trial treatment.  10) Any active or recent history of a known or suspected autoimmune disease or recent history of a syndrome that required systemic corticosteroids (> 10 mg daily prednisone equivalent) or immunosuppressive medications except for syndromes which would not be expected to recur in the absence of an external trigger.  11) Evidence of interstitial lung disease, active non-infectious pneumonitis, or a history of grade 3 or greater pneumonitis.  12) Active infection requiring systemic therapy.  13) History of Human Immunodeficiency Virus (HIV) (HIV 1/2 antibodies).  14) Any positive test for hepatitis B or hepatitis C virus indicating acute or chronic infection.  15) Live vaccine within 30 days prior to the first dose of trial treatment.  16) Chronic, daily treatment with high-dose aspirin (>325 mg/day).  17) Significant vascular disease (e.g., aortic aneurysm requiring surgical repair or recent arterial thrombosis) within 6 months of study enrolment.  18) Any previous venous thromboembolism > NCI CTCAE Grade 3.  19) History of abdominal fistula, GI perforation, intra-abdominal abscess or active GI bleeding within 6 months prior to the first study treatment.  20) Current or recent (within 10 days prior to study treatment start) ongoing treatment with anticoagulants for therapeutic purposes.  21) Major surgical procedure, open biopsy, or significant traumatic injury within 28 days prior to study treatment start, or anticipation of the need for major surgical procedure during the course of the study.  22) Presence of colic prosthesis or stent.  23) History of any severe hypersensitivity reactions to any monoclonal antibody.  24) Women of childbearing potential who are pregnant or breastfeeding. |
